# Modification of the Integrated Sasang Constitutional Diagnostic Model

**DOI:** 10.1155/2017/9180159

**Published:** 2017-11-27

**Authors:** Jiho Nam, Jun-Su Jang, Honggie Kim, Jong Yeol Kim, Jun-Hyeong Do

**Affiliations:** ^1^Medizen Humancare Inc., 20F Keungil Tower, 223 Teheran-ro, Seoul, Republic of Korea; ^2^KM Fundamental Research Division, Korea Institute of Oriental Medicine, 1672 Yuseong-daero, Daejeon, Republic of Korea; ^3^Department of Information and Statistics, Chungnam National University, 99 Daehak-ro, Daejeon, Republic of Korea

## Abstract

In 2012, the Korea Institute of Oriental Medicine proposed an objective and comprehensive physical diagnostic model to address quantification problems in the existing Sasang constitutional diagnostic method. However, certain issues have been raised regarding a revision of the proposed diagnostic model. In this paper, we propose various methodological approaches to address the problems of the previous diagnostic model. Firstly, more useful variables are selected in each component. Secondly, the least absolute shrinkage and selection operator is used to reduce multicollinearity without the modification of explanatory variables. Thirdly, proportions of SC types and age are considered to construct individual diagnostic models and classify the training set and the test set for reflecting the characteristics of the entire dataset. Finally, an integrated model is constructed with explanatory variables of individual diagnosis models. The proposed integrated diagnostic model significantly improves the sensitivities for both the male SY type (36.4% → 62.0%) and the female SE type (43.7% → 64.5%), which were areas of limitation of the previous integrated diagnostic model. The ideas of these new algorithms are expected to contribute not only to the scientific development of Sasang constitutional medicine in Korea but also to that of other diagnostic methods for traditional medicine.

## 1. Introduction

In recent years, the interest in and use of constitutional health care services have been reported to increase consistently. According to a survey by the Korea Institute of Oriental Medicine (KIOM), the percentage of constitutional health services in total traditional medical services reached 30.7% in 2015, which is approximately 7% higher than the corresponding value of 23.8% in 2004 [1].

Sasang constitutional medicine is a personalized medicine that diagnoses the patient's constitution as one of the four Sasang constitutional (SC) types (Tae-Yang: TY, Tae-Eum: TE, So-Yang: SY, and So-Eum: SE) and treats him/her differently depending on the constitution. Therefore, a precise diagnosis of constitution is central to producing consistent results when various experts diagnose the constitution of a single person.

From 2007 to 2011, KIOM collected face, body, voice, and questionnaire data from 2,773 subjects who were diagnosed in terms of constitutional prescriptions by Sasang constitutional experts in 23 oriental medical clinics. Based on these data, an objective and comprehensive Sasang constitutional diagnostic model, which has addressed the existing problems, was proposed in 2012 [2].

Based on this model, many studies on the relationship between Sasang constitution and disease have been conducted. For example, the SY and TE types are independent risk factors for nonalcoholic fatty liver disease [3] regardless of obesity level, TE is a strong risk factor for type 2 diabetes [4], and metabolic syndrome increases the risk of cardiovascular disease in certain physical conditions, such as the SY type [5]. Additionally, physiological characteristics may differ across Sasang types: the SY type has a higher value of total nasal resistance compared to the TE and SE types [6], and the TE type may tolerate psychological or oxidative stress better than the other types [7]. Other studies on constitution-specific physiology, such as comparisons of gut microbiota among Sasang constitutional types [8], have also been conducted using a constitutional diagnostic model.

As increasingly more researchers use the diagnostic model developed by KIOM, multiple issues have also been raised.

Firstly, the ratio of predicted SC types should be the same as the proportions of actual SC types. In the existing model, the predicted proportion of the TE type is much higher than the actual one, which, we believe, is due to the fact that the proportion of TE type is the highest.

Secondly, the multicollinearity problem results from the fact that the explanatory variables of the diagnostic model are highly correlated, making the regression coefficient estimates unreliable.

Finally, in the existing diagnostic model, it was assumed that all individual diagnostic components equally contribute to the diagnosis of the SC types, which turns out to be somewhat unreasonable.

Therefore, in this paper, we propose a novel modified model to solve the problems of the previous diagnostic model, and we compare the revised model with the previous model in terms of various aspects.

## 2. Methods

### 2.1. Participants and Data Acquisition

Using the same standard operating procedure used in the previous study, we collected more face, body shape, voice, and questionnaire data from the subjects than the previous study. A total of 3,849 patients, ranging from teenagers to people in their eighties, were recruited from 23 sites (oriental medical clinics) between November 2006 and August 2012. This process was approved by the KIOM Institutional Review Board (I-0910/02-001) and we obtained written informed consent from the subjects. As in the previous study [2], several patients were excluded for a variety of reasons, such as a small number of TY-type subjects, subjects below the age of 15 having growth spurts, or improper data (Table 1).  Table 2 shows the distribution of subjects by age group.

In comparison with the previous study, the data for one year (August 2011 to August 2012) have been added, and the data extraction algorithm has been enhanced to increase the usage.

### 2.2. Candidate Feature Variables

#### 2.2.1. Facial Images

**Figure 1:**
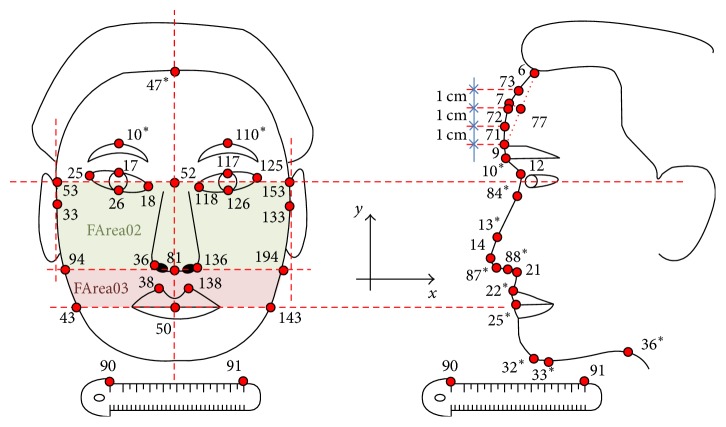
Feature points extracted automatically from facial images. ^*∗*^Added feature point.

A total of 57 facial feature points, including 13 newly added points over the previous study, were extracted using an automatic feature extraction algorithm (Figure 1). However, the feature points of the upper eyelid line, which require a high-resolution image, were excluded for more efficient implementation. Facial candidate feature variables using the extracted points are described in Table 3.

#### 2.2.2. Body Shape

In contrast to the previous study, we excluded both BMI and body weight and reconstructed the model with the remaining variables. The reason for this exclusion is that there is a prejudice to diagnose as a TE type if the BMI and body weight are high and to diagnose as an SE type if BMI and body weight are low. Moreover, in the literature, it is noted that the body shape of the TE type is tall and large rather than obese, and the body shape of the SE type is short and small rather than slender [9].

#### 2.2.3. Voice

Vocal features were extracted using a C++ program combined with the hidden Markov model toolkit [10]. Input vocal signals were divided into multiple windows corresponding to the reference time duration for feature extraction. The window size was 46.4 ms and was mapped to 2^11^ samples at a 44.1 kHz sampling frequency.

The voice signal used was a recording of five vowels and one sentence repeated twice. Unlike the previous study [2], feature extraction was performed only from the sentence, excluding the vowels. Generally, the sentence was more suitable than the vowels because of the unchanged voice information for characterizing an individual. The accuracy and repeatability of the Sasang constitution diagnosis were better in the case of using only the sentence compared to the case of using both the vowels and the sentence [11].

A description of vocal features is provided in Table 4. The harmonic-to-noise ratio (HNR) and the cepstral peak prominence (CPP) are newly added features not found in our previous study [2].

The HNR is a measure that quantifies the amount of additive noise in the voice signal. It is widely used to characterize healthy and disordered voices. The CPP is known to be an accurate predictor and a more reliable measure of dysphonia than other vocal features, such as jitter, shimmer, and HNR [12].

#### 2.2.4. Questionnaire

Binary variables were constructed using the response categories of the questions in the questionnaire, which consisted of multiple-choice questions in Supplementary Table S1 in Supplementary Material available online at https://doi.org/10.1155/2017/9180159. The procedure of generating questionnaire continuous variables is summarized in Supplementary Figure S1.

#### 2.2.5. Compensating for Age Differences

As in previous study, because the candidate feature variables may have shown age-specific trends, a process to eliminate the effect of age was considered by normalizing each candidate feature variable with moving average and standard deviation of the variable for the given age [2].

### 2.3. Model for Sasang Constitutional Diagnosis

#### 2.3.1. Individual Diagnostic Models

The regression coefficients of the explanatory variables used in the previous individual models were estimated using ordinary least squares (OLS). However, the OLS estimator may be acquired with a large variance of the coefficients and be inestimable if the dimension of explanatory variables is too high or each of them is highly intercorrelated. These problems are referred to as overfitting and multicollinearity, respectively. One well-known solution to these problems is the least absolute shrinkage and selection operator (LASSO), which shrinks the variance of the coefficients and makes other coefficients zero [13]. Coefficients were estimated using the glmnet package implemented in the R software. The tuning parameter *λ* was selected from the result of 10-fold cross-validation using the mean square error (MSE) to measure the risk of loss. The decision rule is to choose log⁡(*λ*) that gives the minimum mean cross-validated error.

In the questionnaire model, we calculated questionnaire continuous variables with binary variables using LASSO (Supplementary Table S5) and then we constructed questionnaire model with continuous variables using OLS because the dimension of explanatory variables is low and each of them is not highly intercorrelated.

In addition, because the ratio of SC types is not uniform, the tendency in the previous model was to classify the TE type as the highest ratio. As a result, the sensitivity of TE type was high, but the sensitivity of other SC types was significantly low.

Sensitivity was low, particularly in the male SY type and female SE type. To compensate for this problem, weighting by SC types was added to the model by considering the ratios of SC types.

In the previous model, the training and test sets were separated based on the data collection year. However, there was a problem that the two groups are heterogeneous, which came from the effect of differences due to the collection year. In the present model, the complete set was randomly divided into a training set and a test set at a ratio of 7 : 3 considering SC type and age.

The results of estimated coefficients for face, voice, body shape, and questionnaire continuous features for each SC type are shown in Supplementary Tables S2, S3, S4, and S6, respectively.

#### 2.3.2. Integrating Diagnostic Models from Four Diagnostic Components

Let *π*_*ij*_ be the estimated probability of the *i*th subject in category *j* for each individual diagnostic model, where *j* = 1, 2, and 3 indicate TE, SE, and SY type, respectively.

In the previous study, the importance of each individual diagnostic model was also considered through multiplying the weights by *π*_*ij*_. The integrated estimated probability of an SC type *j* for the *i*th subject, denoted as TSCORE_*ij*_, can be defined by the sum of (*π*_*ij*_)_*r*_ with weight *w*_*r*_:

(1) $$\begin{array}{c} {TSCORE}_{ij}=\overset{4}{\underset{r=1}{\sum }}w_{r}{\left(\;\pi_{ij}\;\right)}_{r}, \end{array}$$

where *r* indicates each individual diagnostic component; *r* = 1, 2, 3, and 4 represent face, body shape, questionnaire, and voice, respectively.

In this study, we constructed a model with explanatory variables as (*π*_*ij*_)_*r*_ of individual diagnosis models. This scheme can be regarded as weighting by a methodical and rigorous method, unlike the case of the previous study, in which arbitrary weights were set.

In addition, compared to the previous weight *w*_*r*_ assigned to each individual diagnostic component, the weight *w*_*jr*_ of this study was obtained considering each component and SC type as shown in Supplementary Table S7:

(2) $$\begin{array}{c} {TSCORE}_{ij}=\overset{4}{\underset{r=1}{\sum }}w_{jr}{\left(\;\pi_{ij}\;\right)}_{r}. \end{array}$$

Finally, the predicted SC type for the *i*th subject was determined in the same way as in the previous study.

(3) Predicted SCi=argmax⁡TSCOREi1,TSCOREi2,TSCOREi3,

where the numbers of the subscripts indicate the TE, SE, and SY types.

## 3. Results

The predicted results of the proposed integrated diagnostic model are shown in Table 5. Relative to the predictions of the previous integrated diagnostic models, the accuracy in the test set is improved by approximately 10% on average. Moreover, the sensitivities of the male SY type (36.4% → 62.0%) and the female SE type (43.7% → 64.5%), which were low in the previous model, were significantly improved, while the sensitivity of the TE type and that of the female SY type were somewhat lowered.

Table 6 shows the results of applying a cutoff to the predicted results of the proposed integrated diagnostic model. To extract more typical SC-type predictions, the reference value for the cutoff criterion was changed to the maximum value minus the second highest value from the maximum value among the three probability values of the SC types, which was proposed in the previous study. This change was determined after implementing various cutoff value transformations.

Comparing the performances before and after the cutoff, the sensitivities and accuracies of each SC type were improved by approximately 7% on average. In particular, the sensitivity of the female SE type improved significantly.

This result alone does not make the comparison with the previous model justified due to the fact that it is not a comparison for the same test set. In this study, the number of data cases is greater and the complete dataset is divided into the training set and test set at random. To make an accurate comparison of the present model with the previous one, an additional comparison was performed with only the common part of the data that were included in finding both models among the test sets used in this model (Table 7).

A comparison of the two results shows that the sensitivities of the previous model are greater for the male TE type and the female SY type, but the sensitivities and accuracies of the other SC types are higher than average by more than 10%.

Although the result of the previous study has an advantage because some training data from the previous study are included in the test set of this study, it is notable that the overall performance of the proposed model is significantly improved.

## 4. Discussion and Conclusions

Relative to the previous studies, the diagnostic performance is significantly improved because of the reasonable considerations of various methodological approaches and the increased number of data cases and variables used to construct the model.

Although the previous diagnostic model offers the same accuracy as the overall model, the sensitivity for the TE type, which has the highest ratio among the SC types, is the highest, whereas the sensitivities for the SE and SY types are significantly lower. This result occurs because the estimation of the regression coefficient of the model is focused on reducing the error for the TE type. Therefore, to improve the performance for the SE and SY constitutional types, although the diagnostic performance for the TE type is somewhat degraded, the regression coefficients of the model are estimated with equal weight by training the model with different weights for each SC type based on their proportions. The TE type, which was frequently predicted in the previous model, was confirmed to have inferior performance, but the performance associated with the other SC types was improved.

As the regression coefficients are difficult to analyze individually because the model consists of multiple explanatory variables, the explanatory variables used in this model are highly correlated because the calculated variables are from similar positions on the body.

In general, the width of a confidence interval of an estimated regression coefficient increases when multicollinearity occurs. The fact that the confidence interval is wide implies that the estimated regression coefficient value is not actually confirmed but is highly likely to differ from the true value of the regression coefficient. Therefore, the influence of the variable cannot be explained correctly, and, as a result, the reliability of the model itself deteriorates.

Among the methods that can solve the multicollinearity problem, LASSO, as used in this study, can reduce multicollinearity without the modification of explanatory variables. Although the resolution of multicollinearity does not lead to improved accuracy, the results suggest that the reliability of the regression coefficient is satisfiable and that stable results will be obtained when new data are analyzed using the model.

In the previous study, the training and test sets were classified based on the data collection year, but the proportions of SC types and age may be heterogeneous in the training and test sets. Therefore, considering the proportions of SC types and age, it is reasonable and well reflective of the characteristics of the entire dataset to obtain both the training set and the test set from the complete dataset.

In traditional medicine in Asia, including Chinese medicine, facial, body shape, voice, and pulse information, as well as ordinary symptoms, are combined in the process of diagnosis for prescription decisions. In particular, many studies on the relationship between* Prakriti* (Ayurveda constitution) and various objective biological parameters have been actively conducted in the field of* Ayurveda*, and, at the same time, efforts are being made to propose a standard protocol for the objective and reliable diagnosis of* Prakriti* [14]. We believe that the proposed method is the most advanced tool to support the ability of Asian doctors to diagnose in an objective and scientific way. The improvement of this algorithm is expected to contribute not only to the scientific development of Sasang constitutional medicine in Korea but also to that of other traditional medical diagnosis methods.

## Supplementary Material

Table S1. List of Questions.
Table S2. Result of estimated coefficients for Facial features for each SC type using LASSO.
Table S3. Result of estimated coefficients for Voice features for each SC type using LASSO.
Table S4. Result of estimated coefficients for Body shape features for each SC type using LASSO.
Table S5. Result of estimated coefficients for Questionnaire features for each SC type using LASSO.
Table S6. Result of estimated coefficients for Questionnaire continuous features for each SC type using OLS.
Table S7. Result of estimated coefficients for estimated probability of individual diagnostic model for each SC type using LASSO.
Figure S1. The procedure of generating continuous variables with the response categories of the questions in the questionnaire.

## Figures and Tables

**Table 1:** The number of excluded and included data cases for each individual diagnostic model.

Reason for exclusion (total number of subjects: 3,849)	Face	Body shape	Voice	Questionnaire
Not collected initially	182	0	592	624
TY constitution	76	76	73	73
Below 15 years of age	96	107	79	79
Data extraction errors and missing cases	1,343	429	388	150
Final number of subjects	2,152	3,237	2,717	2,923
Common samples after integrating the four components (for the integrated diagnostic model)	1,891 (male: 657; female: 1,234)			

**Table 2:** Distribution of subjects by age group.

Age group	Face		Body shape		Voice		Questionnaire		Common samples					
									TE		SE		SY	
	M^*∗*^	F^*∗∗*^	M	F	M	F	M	F	M	F	M	F	M	F
10s	32	29	50	41	45	40	46	41	10	14	13	6	6	8
20s	89	162	120	234	97	198	98	214	23	45	27	50	25	48
30s	131	273	186	375	159	326	165	346	46	66	35	81	32	93
40s	161	317	245	453	202	381	214	416	58	109	42	73	41	95
50s	169	317	264	464	219	375	240	412	61	99	34	72	49	105
60s~	171	301	299	506	244	431	267	464	66	128	22	68	67	74

Total	2,152		3,237		2,717		2,923		1,891					

^*∗*^M: male; ^*∗∗*^F: female.

**Table 3:** Candidate facial feature variables.

		Size-related variables		Shape-related variables
Face shape	(i) Width	FD_43_143, FD_53_153, FD_94_194, FDH_33_133	(i) Angle	FA_53_94, FAs_153_194, FA_94_43, FAs_194_143, FA_53_94_43, FA_18_25_43, FA_118_125_143,
	(ii) Depth	PDH_44_53, PDH_12_36, PDH_14_36, PDH_21_36		FA_18_17_43, FA_118_117_143, FA_17_25_43, FA_117_125_143, FA_18_25_94, FA_18_43_50,
				FA_18_94_50
	(iii) Height	FDV_47_52, FDV_47_50, FDV_10_21, FDV_52_50, FDV_81_50,	(ii) Ratio	FHD_33_133_43_143, FDD_53_153_43_143, FDD_94_194_43_143, FHD_33_133_53_153,
		PDV_6_12, PDV_6_32, PDV_10_32, PDV_12_32, PDV_21_32, PDV_25_32		FVV_47_52_52_81, FVV_47_52_52_50, FVV_47_52_81_50, FVV_52_81_81_50, PVV_6_10_10_33,
	(iv) Area	FArea02, FArea03, FArea02/FArea03		PVV_12_14_14_32, PVV_12_21_14_32, FVD_52_50_53_153, FVD_52_81_53_153, FVD_81_50_94_194

Forehead	Height:	FDV_47_10, PDV_6_9, PDV_6_10	(i) Angle	PAi_7_6, PAi_9_7, PAi_71_72, PAi_72_73, PA_6_7_9
			(ii) Ratio	PDD_77_9_6_9
			(iii) Depth	PDH_6_7, PDH_9_12
			(iv) Distance	PD_7_77, PDV_6_7, PDV_7_9, PDV_9_12, PA_9_12, PA_10_12

Eye	(i) Width	FDH_18_25, FDH_118_125, FDH_18_118, FDH_25_125, FDH_21_121	(i) Angle	FA_18_17_25, FA_118_117_125, FAi_25_17, FAis_125_117, FAis_18_17, FAi_118_117,
				FAis_18_25, FAi_118_125
	(ii) Height	FD_17_26, FD_117_126	(ii) Ratio	FDH_17_26_18_25, FDH_117_126_118_125, FDH_52_50_18_118, FDD_17_26_52_81, FDD_17_26_52_50,
	(iii) Distance	FD_17_25, FD_117_125, FD_18_25, FD_118_125		(FDH_18_25+FDH_118_125)/2/FDH_18_118, (FDH_18_25+FDH_118_125)/2/FD_53_153,
				(FDH_18_25+FDH_118_125)/2/FDH_33_133, FHD_18_118_53_153, FHD_25_125_53_153

Nose	(i) Width	FDH_36_136, PDH_12_14, PDL_14_12_21	(i) Angle	PAi_14_12, PA_14_21, PA_12_14_21, PAi_13_84, PA_87_88, PA_87_21
	(ii) Height	FDV_52_81, PDV_12_14, PDV_14_21, PDV_12_21, PD_12_21		
	(iii) Depth	PDH_41_21	(ii) Ratio	FHD_36_136_53_153, FVH_52_81_36_136
	(iv) Area	FArea_52_36_136, PArea_12_14_21		

Mouth	(i) Width	PDL_22_21_32, PDL_25_21_32	Ratio	FVV_80_50_52_50, FVV_80_50_81_50
	(ii) Height	FDV_80_50		

Chin	(i) Depth	PDH_32_36	Angle	PA_32_33, PA_33_36, PA_32_33_36
	(ii) Height	PDV_32_36		
	(iii) Distance	PD_32_36		

*Note*. FD(*n*_1_, *n*_2_) [or PD(*n*_1_, *n*_2_)]: distance between points *n*_1_ and *n*_2_ in a frontal (or profile) image;

FDH(*n*_1_, *n*_2_) [or PDH(*n*_1_, *n*_2_)]: horizontal distance between *n*_1_ and *n*_2_ in a frontal (or profile) image;

FDV(*n*_1_, *n*_2_) [or PDV(*n*_1_, *n*_2_)]: vertical distance between *n*_1_ and *n*_2_ in a frontal (or profile) image;

FA(*n*_1_, *n*_2_) [or PA(*n*_1_, *n*_2_)]: angle between the line through two points *n*_1_ and *n*_2_ and a horizontal line in a frontal (or profile) image;

FA(*n*_1_, *n*_2_, *n*_3_) [or PA(*n*_1_, *n*_2_, *n*_3_)]: angle between three points, *n*_1_, *n*_2_, and *n*_3_, in a frontal (or profile) image;

PAR(*n*_1_, *n*_2_, *n*_3_): area of the triangle formed by three points, *n*_1_, *n*_2_, and *n*_3_, in a profile image;

FHD_*n*_1__*n*_2__*n*_3__*n*_4_ [or PHD_*n*_1__*n*_2__*n*_3__*n*_4_] = FDH_*n*_1__*n*_2_/FD_*n*_3__*n*_4_ [or PDH_*n*_1__*n*_2_/PD_*n*_3__*n*_4_];

FDH_*n*_1__*n*_2__*n*_3__*n*_4_ [or PDH_*n*_1__*n*_2__*n*_3__*n*_4_] = FD_*n*_1__*n*_2_/FDH_*n*_3__*n*_4_ [or PD_*n*_1__*n*_2_/PDH_*n*_3__*n*_4_];

FDD_*n*_1__*n*_2__*n*_3__*n*_4_ [or PDD_*n*_1__*n*_2__*n*_3__*n*_4_] = FD_*n*_1__*n*_2_/FD_*n*_3__*n*_4_ [or PD_*n*_1__*n*_2_/PD_*n*_3__*n*_4_];

FVD_*n*_1__*n*_2__*n*_3__*n*_4_ [or PVD_*n*_1__*n*_2__*n*_3__*n*_4_] = FDV_*n*_1__*n*_2_/FD_*n*_3__*n*_4_ [or PDV_*n*_1__*n*_2_/PD_*n*_3__*n*_4_];

FVV_*n*_1__*n*_2__*n*_3__*n*_4_ [or PVV_*n*_1__*n*_2__*n*_3__*n*_4_] = FDV_*n*_1__*n*_2_/FDV_*n*_3__*n*_4_ [or PDV_*n*_1__*n*_2_/PDV_*n*_3__*n*_4_];

FVH_*n*_1__*n*_2__*n*_3__*n*_4_ [or PVH_*n*_1__*n*_2__*n*_3__*n*_4_] = FDV_*n*_1__*n*_2_/FDH_*n*_3__*n*_4_ [or PDV_*n*_1__*n*_2_/PDH_*n*_3__*n*_4_].

**Table 4:** Description of sentence features.

Sentence feature^*∗*^	Description
sF0, sFCV	Average pitch frequency and coefficient of variation of the pitch
sF10, sF50, sF90	10th, 50th, and 90th percentiles of pitch distribution
sFHL	Ratio of (sF90-sF50) to (sF50-sF10)
sDT	Duration time of a sentence reading
sHNR	HNR
sCPP	CPP
sMFCC1~12	12 Mel-frequency cepstral coefficients

^*∗*^All feature values are based on the averaged output of the two sentence utterances, which were repeated recordings of the same sentence.

**Table 5:** Diagnostic results of the proposed integrated diagnostic model.

	Male					Female				
	Predicted SC type				Sensitivity	Predicted SC type				Sensitivity
	TE	SE	SY	Total		TE	SE	SY	Total	
Training set										
True SC type										
TE	144	14	30	188	76.6%	223	32	66	321	69.5%
SE	14	81	26	121	66.9%	33	146	63	242	60.3%
SY	29	28	92	149	61.7%	65	65	168	298	56.4%
Total	187	123	148	458		321	243	297	861	
Accuracy	69.2%					62.4%				

Test set										
True SC type										
TE	64	4	8	76	84.2%	104	12	25	141	73.8%
SE	8	35	9	52	67.3%	12	69	26	107	64.5%
SY	22	5	44	71	62.0%	25	28	72	125	57.6%
Total	94	44	61	199		141	109	123	373	
Accuracy	71.9%					65.7%				

**Table 6:** Diagnosis results of proposed integrated diagnosis model using a cutoff.

	Male					Female				
	Predicted SC type				Sensitivity	Predicted SC type				Sensitivity
	TE	SE	SY	Total		TE	SE	SY	Total	
Training set										
True SC type										
TE	131	9	19	159	82.4%	181	15	39	235	77.0%
SE	10	71	17	98	72.4%	19	110	28	157	70.1%
SY	19	15	68	102	66.7%	39	40	114	193	59.1%
Total	160	95	104	359		239	165	181	585	
Accuracy	75.2%					69.2%				

Test set										
True SC type										
TE	58	3	3	64	90.6%	89	11	14	114	78.1%
SE	4	29	5	38	76.3%	8	58	9	75	77.3%
SY	15	4	38	57	66.7%	12	14	49	75	65.3%
Total	77	36	46	159		109	83	72	264	
Accuracy	78.6%					74.2%				

**Table 7:** Comparison of diagnosis results of the set of common data between the test set of the proposed integrated diagnosis model and the full set of the previous integrated diagnosis model.

	Male					Female				
	Predicted SC type				Sensitivity	Predicted SC type				Sensitivity
	TE	SE	SY	Total		TE	SE	SY	Total	
Proposed integrated diagnosis model										
True SC type										
TE	36	2	5	43	83.7%	68	9	16	93	73.1%
SE	3	21	8	32	65.6%	7	46	15	68	67.6%
SY	14	4	24	42	57.1%	13	21	47	81	58.0%
Total	53	27	37	117		88	76	78	242	
Accuracy	69.2%					66.5%				

Previous integrated diagnosis model										
True SC type										
TE	38	3	2	43	88.4%	62	6	25	93	66.7%
SE	9	15	8	32	46.9%	10	27	31	68	39.7%
SY	15	7	20	42	47.6%	18	10	53	81	65.4%
Total	62	25	30	117		90	43	109	242	
Accuracy	62.4%					58.7%				
