# Supplementary material for: Modification of the Integrated Sasang Constitutional Diagnostic Model
Source: Evid Based Complement Alternat Med. 2017 Nov 27;2017:9180159. doi: 10.1155/2017/9180159 (PMC5727843; doi:10.1155/2017/9180159)

Table S1. List of Questions

Personality Questionnaire					
This survey asks for your views about your personality. This information will be help keep track of your constitution. Answer every question by marking (✔) the answer as indicated. If you are unsure about how to answer a question, please give the best answer you can. ② means the middle status.					
1. Are you broad-minded or delicate?	broad-minded	①	②	③	delicate
2. Do you tend to act quickly or slowly?	quickly	①	②	③	slowly
3. Do you tend be active or passive?	active	①	②	③	passive
4. When expressing your opinion, do you tend to do it straightly or indirectly?	directly	①	②	③	indirectly
5. Do you tend to be extrovert or introvert?	extrovert	①	②	③	introvert
6. Do you tend to be energetic or quiet?	energetic	①	②	③	quiet
7. Is it easy or hard for you to make quick decisions?	easy	①	②	③	hard
8. Do you tend to be masculine or feminine?	masculine	①	②	③	feminine
9. Do you tend to discuss or hide your private life from others?	open	①	②	③	hide
10. Do you tend to express your opinions or keep them inside?	express	①	②	③	keep inside
11. Do you tend to be excited or rational when you deal something?	excited	①	②	③	rational
12. Do you tend to act hastily or carefully?	hastily	①	②	③	carefully

Questionnaire on Habits	
Please check your habits within 6 months based on your usual life style. You can ask the person in charge when the question is ambiguous. You can check more than twice for the questions with *.	
**Meal**	13. How much do you eat daily?<br>1☐ much 2☐ medium 3☐ a little 4☐irregular
	14. How fast do you eat?<br>1☐ fast 2☐ medium 3☐ slowly
**Digestion**	15. Do you digest well?<br>1☐yes 2☐no<br>if No --> Does digestive dysfunction upset you? 1☐yes 2☐no
	16. How is your appetite?<br>1☐ Extremely good 2☐good 3☐ medium 4☐ not good
	Do you suffer from these signs?

Questionnaire on Habits

Signs	Regularly	Some time	Never
17. Epigastric discomfort	1☐	2☐	3☐
18. Vomiting	1☐	2☐	3☐
19. Indigestion	1☐	2☐	3☐

Perspiration

20. Generally, how much do you perspire? 1☐a lot 2☐moderate 3☐little 4☐Never

21. In hot weather, how much do you perspire? 1☐a lot 2☐moderate 3☐little 4☐Never

22. How do you feel after perspiration? 1☐refreshed 2☐tired 3☐no feeling

23. Do you have cold sweat or perspire in below situations? (You can check several times)
1☐never 2☐sweat at night 3☐sweat during day 4☐sweat during meals

Defecation

24. How about the condition of your stool?
1☐Diarrhea 2☐Constipation 3☐Diarrhea and constipation alternatively
4☐like indigested food 5☐Normal

25. What is the shape of your stool? Chose the most appropriate shape.

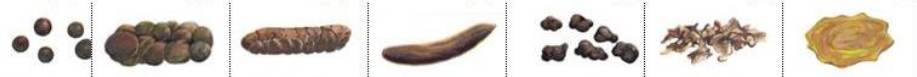

1☐	2☐	3☐	4☐	5☐	6☐	7☐
Hard, dry, round, and small shape	Hard, compact, and sausage-shaped	Sausage-shaped with surface cracks across the surface	Soft and Ice-cream shaped	Soft with chunks	Porridge consistency and scattered	Watery diarrhea without solids

Sleep

26. Your real sleeping time per day is ☐☐ hours and ☐☐ minutes
(real sleeping time refers to the sleeping duration not the duration staying in bed)

Heat and cold

27. In general, do you dislike cold or heat? 1☐cold 2☐heat 3☐both 4☐both are ok

28. Your hands always are 1☐ warm 2☐ medium 3☐ cold 4☐ not sure

29. Your feet always are 1☐ warm 2☐ medium 3☐ cold 4☐ not sure

30. Your abdomen always are 1☐ warm 2☐ medium 3☐ cold 4☐ not sure

Drinking water

31. How much water do you drink a day?
1☐a lot 2☐medium 3☐a little 4☐frequently with small amount

32. How is the temperature of the water you drink?
1☐usually hot water 2☐usually cold water 3☐ not specific

Catching common cold

33. If you catch a cold, which of the following symptoms do you have?
(You can check several times)
1☐no symptoms 2☐fever 3☐headache 4☐dizzy
5☐runny or stuffy nose
6☐loss of hearing 7☐cough(phlegm) 8☐sore throat 9☐body pain

Questionnaire on Habits	
	10☐numbness in my arms and legs 11☐loss of appetite and/or indigestion
Condition	34. Which of the following do you experience when not feeling healthy?<br>(You can check several times)<br>1☐sweating 2☐indigestion 3☐bowel issues 4☐urinary issues<br>5☐not specific
Other	35. Do you experience any of the symptoms below?<br>(You can check several times)<br>1☐lateral sides of the head discomfort 2☐feverish headache<br>3☐discomfort at the head with empty feeling<br>4☐loss of memory 5☐mouth frequently blistered 6☐eye pain 7☐swelling<br>8☐palpitation<br>9☐shortness of breath 10☐frequent sigh 11☐dizziness 12☐none of the above

Table S2. Result of estimated coefficients for Facial features for each SC type using LASSO

Features	Male			Female		
	$\hat{\beta}_{1,lasso}(\hat{\lambda})$	$\hat{\beta}_{2,lasso}(\hat{\lambda})$	$\hat{\beta}_{3,lasso}(\hat{\lambda})$	$\hat{\beta}_{1,lasso}(\hat{\lambda})$	$\hat{\beta}_{2,lasso}(\hat{\lambda})$	$\hat{\beta}_{3,lasso}(\hat{\lambda})$
$\hat{\beta}_{0,lasso}(\hat{\lambda})$	-0.03	0.01	0.02	-0.21	-0.06	0.27
AGE		0.00	0.00	0.00		0.00
FDV_47_52						
FDV_47_50						
FDV_10_21		0.03	-0.06	-0.08	0.01	
FDV_52_50		-0.03				
FDV_52_81				0.36		
FDV_81_50						
FD_43_143_aD	0.07				-0.24	
FD_53_153_aD					-0.13	
FD_94_194_aD		-0.01				
FDH_33_133_aD					-0.18	
FHD_33_133_43_143		0.01	-0.41			
FDD_53_153_43_143						
FDD_94_194_43_143						
FHD_33_133_53_153			-0.01	-0.29		
FDD_94_194_53_153						
FVV_47_52_52_81					0.25	
FVV_47_52_52_50						
FVV_47_52_81_50	-0.14	0.23				
FVV_52_81_81_50						
FVD_52_50_53_153_aD					0.07	
FVD_52_81_53_153_aD	-0.55					0.00
FVD_81_50_94_194_aD				-0.42	0.15	
FA_53_94				0.12	-0.01	
FAs_153_194	0.16		-0.39	0.26	-0.25	
FA_94_43				0.05		
FAs_194_143	-0.02	0.00		0.03		
FA_53_94_43		0.16				
FA_18_25_43			-0.16			
FA_118_125_143	0.10	-0.23			0.19	
FA_18_17_43	0.04					

FA_118_117_143	0.18	-0.30				
FA_17_25_43						-0.13
FA_117_125_143				-0.03		
FA_18_25_94				0.13		
FA_18_43_50	-0.15				0.01	
FA_18_94_50		0.09				
FArea02_aD	0.39					
FArea03_aD					0.01	-0.08
FArea02_03	0.06			-0.29	0.18	
FDV_47_10				0.03	-0.07	
FDH_18_25						
FDH_118_125			-0.05			
FDH_18_118						
FDH_25_125						-0.18
FDH_21_121		-0.23				
FD_17_26					-0.14	0.03
FD_117_126	0.19	-0.11				
FD_17_25		-0.30	0.04			
FD_117_125			-0.14	0.19		
FD_18_25						
FD_118_125				0.10		
FDH_17_26_18_25						
FDH_117_126_118_125					0.09	-0.15
FDH_52_50_18_118						
FRE_H01			0.18		0.04	-0.08
FRE_H02_aD						
FRE_H05_aD						
FHD_18_118_53_153_aD		0.11				
FHD_25_125_53_153_aD				-0.05	0.02	
FDD_17_26_52_81		0.20				
FDD_17_26_52_50		0.00				0.10
FA_18_17_25			-0.06		-0.28	
FA_118_117_125				0.04		-0.14
FAi_25_17	-0.12				0.02	

FAis_125_117	-0.05	0.01		-0.03		0.05
FAis_18_17		-0.30	0.02			-0.09
FAi_118_117						
FAis_18_25						
FAi_118_125		-0.20				
FDH_36_136						
FHD_36_136_53_153_aD	0.00			0.00	0.09	
FVH_52_81_36_136	0.20					
FArea_52_36_136			-0.04			0.00
FDV_80_50	0.15			0.22	-0.01	
FVV_80_50_52_50						
FVV_80_50_81_50	-0.18	0.23			-0.06	
PD_7_77	0.03				-0.04	
PDV_7_9		-0.13	0.10		-0.02	0.07
PAi_9_7	0.02		-0.04			0.00
PAi_71_72				0.01	-0.04	
PAi_72_73	0.00	0.22		-0.04	0.01	
PDV_9_12	0.31	-0.16		0.25		-0.05
PDH_9_12					-0.28	0.19
PA_9_12			-0.18		-0.19	0.01
PA_10_12	-0.32		0.10	-0.15		0.16
PDH_12_14			-0.26	0.05		-0.21
PDH_14_21					-0.15	0.05
PDL_14_12_21						
PDV_12_14					0.02	
PDV_14_21		-0.07				
PDV_12_21		-0.26			0.05	
PD_12_21						
PArea_12_14_21			-0.06			
PAi_14_12		-0.01				
PA_14_21						
PA_12_14_21		-0.04	0.03			
PAi_13_84	0.06	0.00		0.05		
PA_87_21		0.00	-0.21			0.02

$\hat{\beta}_{1,lasso}(\acute{\lambda}), \hat{\beta}_{2,lasso}(\acute{\lambda}), \hat{\beta}_{3,lasso}(\acute{\lambda})$ represent result of estimated coefficients via LASSO for each SC group, TE, SE, and SY, respectively, where $\acute{\lambda} = e^{\log(\acute{\lambda})}$, chosen from the result of ten-fold cross validation.

Table S3. Result of estimated coefficients for Voice features for each SC type using LASSO

Features	Male			Female		
	$\hat{\beta}_{1,lasso}(\acute{\lambda})$	$\hat{\beta}_{2,lasso}(\acute{\lambda})$	$\hat{\beta}_{3,lasso}(\acute{\lambda})$	$\hat{\beta}_{1,lasso}(\acute{\lambda})$	$\hat{\beta}_{2,lasso}(\acute{\lambda})$	$\hat{\beta}_{3,lasso}(\acute{\lambda})$
$\hat{\beta}_{0,lasso}(\acute{\lambda})$	0.10	0.02	-0.12	-0.47	0.25	0.22
AGE		0.00	0.00	0.02		0.00
sF10	0.11	-0.38		0.17		-0.15
sF50	-0.46	0.04			0.19	-0.42
sF90			0.23		0.40	-0.44
sFHL	-0.20	0.20			-0.06	0.08
sF0	0.17		-0.37		-0.26	0.95
sFCV		-0.22	0.00		-0.13	0.12
sDT		-0.01	0.06	0.02	-0.02	
sMFCC1	0.11		-0.25	0.04	0.00	
sMFCC2		-0.16	0.15		0.21	-0.08
sMFCC3		0.01	-0.18	-0.15	0.10	
sMFCC4		-0.03	0.07	0.02	-0.09	
sMFCC5	-0.06	0.17		0.05	-0.08	
sMFCC6		0.25	-0.12		-0.03	0.09
sMFCC7		0.24	-0.23	-0.05	0.19	
sMFCC8	0.12		-0.20	0.18		-0.20
sMFCC9	0.21	-0.16		0.22	-0.08	
sMFCC10		-0.20	0.13	0.02		-0.01
sMFCC11	0.01	-0.18		-0.12	0.04	
sMFCC12		-0.11		-0.13		0.11
sHNR35		0.01	-0.18		-0.02	0.10
sCPP	0.00		0.08		0.01	-0.06

$\hat{\beta}_{1,lasso}(\acute{\lambda}), \hat{\beta}_{2,lasso}(\acute{\lambda}), \hat{\beta}_{3,lasso}(\acute{\lambda})$ represent result of estimated coefficients via LASSO for each SC group, TE, SE, and SY, respectively, where $\acute{\lambda} = e^{\log(\acute{\lambda})}$, chosen from the result of ten-fold cross validation.

Table S4. Result of estimated coefficients for Body shape features for each SC type using LASSO

Features	Male			Female		
	$\hat{\beta}_{1,lasso}(\acute{\lambda})$	$\hat{\beta}_{2,lasso}(\acute{\lambda})$	$\hat{\beta}_{3,lasso}(\acute{\lambda})$	$\hat{\beta}_{1,lasso}(\acute{\lambda})$	$\hat{\beta}_{2,lasso}(\acute{\lambda})$	$\hat{\beta}_{3,lasso}(\acute{\lambda})$
$\hat{\beta}_{0,lasso}(\acute{\lambda})$	0.02	0.17	-0.19	-0.26	-0.08	0.34
AGE		-0.01	0.01	0.01	0.00	
HEIGHT	-0.14	0.06			0.18	-0.09
WEIGHT						
BMI						
FC	1.12		-0.47	-0.10		0.29
NC	-0.52		0.82	0.56	-1.41	
AC				0.51		-2.77
CC				-0.28		1.84
RC						
WC				0.00		-0.07
HC		-0.56				
NC/FC	0.71			0.00		0.18
AC/FC	0.62		-0.12	0.00		2.07
CC/FC				-0.30	0.00	
RC/FC		0.15	-0.39	0.00	-0.17	
WC/FC				0.53		-0.13
HC/FC	0.21				0.76	-1.10
AC/NC						-2.45
CC/NC	-0.22		0.10	1.04	-0.41	
RC/NC				-1.02		
WC/NC	-0.19		0.15	-0.76		1.58
HC/NC		-0.02			-1.59	0.87
CC/AC	-0.25		0.19	1.44	-0.17	
RC/AC					2.25	-0.19
WC/AC					2.64	-0.55
HC/AC	1.03		-0.02	0.33	-1.95	
RC/CC	-0.64	0.18			0.82	-0.26
WC/CC	1.23		-0.83	1.12	-1.80	
HC/CC	-0.75		1.07		-0.95	0.04
WC/RC	-0.70		0.03	-1.01	0.33	
HC/RC	-0.01	0.99			3.54	-0.70
HC/WC			-0.76	-1.03	0.51	

$\hat{\beta}_{1,lasso}(\acute{\lambda}), \hat{\beta}_{2,lasso}(\acute{\lambda}), \hat{\beta}_{3,lasso}(\acute{\lambda})$ represent result of estimated coefficients via LASSO for each SC group, TE, SE, and SY, respectively, where $\acute{\lambda} = e^{\log(\acute{\lambda})}$, chosen from the result of ten-fold cross validation.

Table S5. Result of estimated coefficients for Questionnaire features for each SC type using LASSO

Features	Male			Female		
	$\hat{\beta}_{1,lasso}(\acute{\lambda})$	$\hat{\beta}_{2,lasso}(\acute{\lambda})$	$\hat{\beta}_{3,lasso}(\acute{\lambda})$	$\hat{\beta}_{1,lasso}(\acute{\lambda})$	$\hat{\beta}_{2,lasso}(\acute{\lambda})$	$\hat{\beta}_{3,lasso}(\acute{\lambda})$
$\hat{\beta}_{0,lasso}(\acute{\lambda})$	0.20	-0.27	0.07	0.34	-0.26	-0.08
Q_1_1		-0.24			-0.28	
Q_1_2						
Q_1_3		0.26			0.23	
Q_2_1	-0.66		0.26	-0.36		0.09
Q_2_2						
Q_2_3						
Q_3_1					-0.29	
Q_3_2						
Q_3_3						-0.05
Q_4_1						
Q_4_2						
Q_4_3						-0.02
Q_5_1		-0.02	0.07			
Q_5_2						
Q_5_3						-0.01
Q_6_1		-0.28				
Q_6_2						
Q_6_3		0.28			0.11	-0.21
Q_7_1		-0.15		-0.15		
Q_7_2						
Q_7_3						
Q_8_1		-0.10		0.03		
Q_8_2						0.04
Q_8_3					0.14	
Q_9_1						0.08
Q_9_2						
Q_9_3				0.06		-0.07
Q_10_1						
Q_10_2			-0.21		-0.07	
Q_10_3						
Q_11_1		-0.12	0.00		-0.23	
Q_11_2						0.00
Q_11_3						
Q_12_1						
Q_12_2						

Q_12_3		0.04			0.02	
Q_13_1				0.22	-0.17	
Q_13_2						
Q_13_3						
Q_13_4				0.22	-0.19	
Q_14_1	0.06			0.17	-0.11	
Q_14_2						
Q_14_3				-0.19		
Q_15_1					-0.13	
Q_15_2					0.21	
Q_15_3						
Q_16_1				0.20		
Q_16_2	0.12	-0.13		0.01	-0.19	
Q_16_3	-0.11			-0.03		
Q_16_4						
Q_17_1				-0.09		
Q_17_2				0.03		-0.05
Q_17_3						
Q_18_1					-0.18	
Q_18_2						
Q_18_3					0.18	
Q_19_1						
Q_19_2						
Q_19_3			-0.04			
Q_20_1		-0.19		0.20	-0.24	
Q_20_2						
Q_20_3	-0.22	0.21				
Q_20_4				-0.07		0.09
Q_21_1		-0.01				
Q_21_2				0.02		
Q_21_3					0.43	
Q_21_4						
Q_22_1		-0.09		0.08	-0.23	
Q_22_2	-0.18				0.31	
Q_22_3				-0.20		
Q_23_1						
Q_23_2						
Q_23_3						
Q_23_4					-0.31	
Q_24_1					0.05	
Q_24_2				0.01		

Q_24_3						
Q_24_4				-0.25		0.05
Q_24_5						
Q_25_1					-0.03	
Q_25_2				0.06		
Q_25_3						-0.19
Q_25_4						0.02
Q_25_5						-0.30
Q_25_6						
Q_25_7						
Q_26_1				-0.04	0.03	
Q_26_2	-0.01	0.00				
Q_27_1	-0.11	0.17				
Q_27_2	0.21	-0.23		0.06		
Q_27_3						
Q_27_4						
Q_28_1	0.26	-0.07		0.16		
Q_28_2						
Q_28_3	-0.03	0.06		-0.53		0.03
Q_28_4					0.50	
Q_29_1						
Q_29_2						
Q_29_3	-0.02		0.03			
Q_29_4						
Q_30_1						
Q_30_2	-0.04					
Q_30_3						-0.13
Q_30_4						
Q_31_1				0.20	-0.15	
Q_31_2						
Q_31_3						
Q_31_4						0.16
Q_32_1		0.13		-0.05	0.37	
Q_32_2						
Q_32_3					-0.15	
Q_33_1					-0.39	
Q_33_2						
Q_33_3	-0.04				0.01	
Q_33_4				-0.03	0.15	
Q_33_5						
Q_33_6						

Q_33_7				0.10	-0.03	
Q_33_8				-0.03		0.03
Q_33_9	0.00		0.05			
Q_33_10					-0.01	
Q_33_11						
Q_34_1				0.04		
Q_34_2		0.90			0.22	
Q_34_3				0.00	-0.19	
Q_34_4						
Q_34_5						
Q_35_1			-0.24	0.04	-0.27	
Q_35_2						
Q_35_3					-0.21	
Q_35_4		-0.14				0.10
Q_35_5						0.12
Q_35_6						
Q_35_7		-0.14		0.47		
Q_35_8						
Q_35_9				-0.16		
Q_35_10						
Q_35_11						
Q_35_12						-0.25

$\hat{\beta}_{1,lasso}(\acute{\lambda}), \hat{\beta}_{2,lasso}(\acute{\lambda}), \hat{\beta}_{3,lasso}(\acute{\lambda})$ represent result of estimated coefficients via LASSO for each SC group, TE, SE, and SY, respectively, where $\acute{\lambda} = e^{\log(\acute{\lambda})}$, chosen from the result of ten-fold cross validation.

Table S6. Result of estimated coefficients for Questionnaire continuous features for each SC type using OLS

SC type	Features	Male			Female		
		β	S.E	p	β	S.E	p
TE	Intercept	0.098	0.590	0.999	-0.350	0.429	0.414
	AGE	0.002	0.012	0.884	0.009	0.009	0.282
	Q_{TE}	0.648	0.228	0.005	0.785	0.146	0.000
	Q_{SE}	-0.183	0.193	0.343	-0.084	0.142	0.556
	Q_{SY}	-0.453	0.210	0.031	-0.513	0.149	0.001
SE	Intercept	-0.600	0.625	0.337	-0.420	0.432	0.330
	AGE	-0.003	0.012	0.806	0.001	0.009	0.881
	Q_{TE}	-0.146	0.227	0.519	-0.004	0.149	0.979
	Q_{SE}	0.933	0.199	0.000	0.913	0.149	0.000
	Q_{SY}	-0.501	0.220	0.023	-0.418	0.146	0.004

Reference category : SY S.E : standard error of β

Table S7. Result of estimated coefficients for estimated probability of individual diagnostic model for each SC type using LASSO

Features	Male			Female		
	$\hat{\beta}_{1,lasso}(\acute{\lambda})$	$\hat{\beta}_{2,lasso}(\acute{\lambda})$	$\hat{\beta}_{3,lasso}(\acute{\lambda})$	$\hat{\beta}_{1,lasso}(\acute{\lambda})$	$\hat{\beta}_{2,lasso}(\acute{\lambda})$	$\hat{\beta}_{3,lasso}(\acute{\lambda})$
$\hat{\beta}_{0,lasso}(\acute{\lambda})$	0.38	0.08	-0.46	0.30	-0.39	0.09
$(\pi_{i1})_1$	0.98			1.13		
$(\pi_{i2})_1$	-0.03	2.42			1.13	
$(\pi_{i3})_1$		-0.09	2.91			1.55
$(\pi_{i1})_2$	3.04		-0.07	2.27		
$(\pi_{i2})_2$	-0.61	0.87		-0.47	2.21	
$(\pi_{i3})_2$		-1.32	0.44			
$(\pi_{i1})_3$	1.17	-0.20		1.09		
$(\pi_{i2})_3$		3.09	-0.40		1.75	
$(\pi_{i3})_3$	-1.42		1.16			2.02
$(\pi_{i1})_4$			-0.65	0.92		
$(\pi_{i2})_4$					2.06	
$(\pi_{i3})_4$		-0.44	2.40			2.36

$\hat{\beta}_{1,lasso}(\acute{\lambda}), \hat{\beta}_{2,lasso}(\acute{\lambda}), \hat{\beta}_{3,lasso}(\acute{\lambda})$ represent result of estimated coefficients via LASSO for each SC group, TE, SE, and SY, respectively, where $\acute{\lambda} = e^{\log(\acute{\lambda})}$, chosen from the result of ten-fold cross validation.

Figure S1. The procedure of generating continuous variables with the response categories of the questions in the questionnaire.

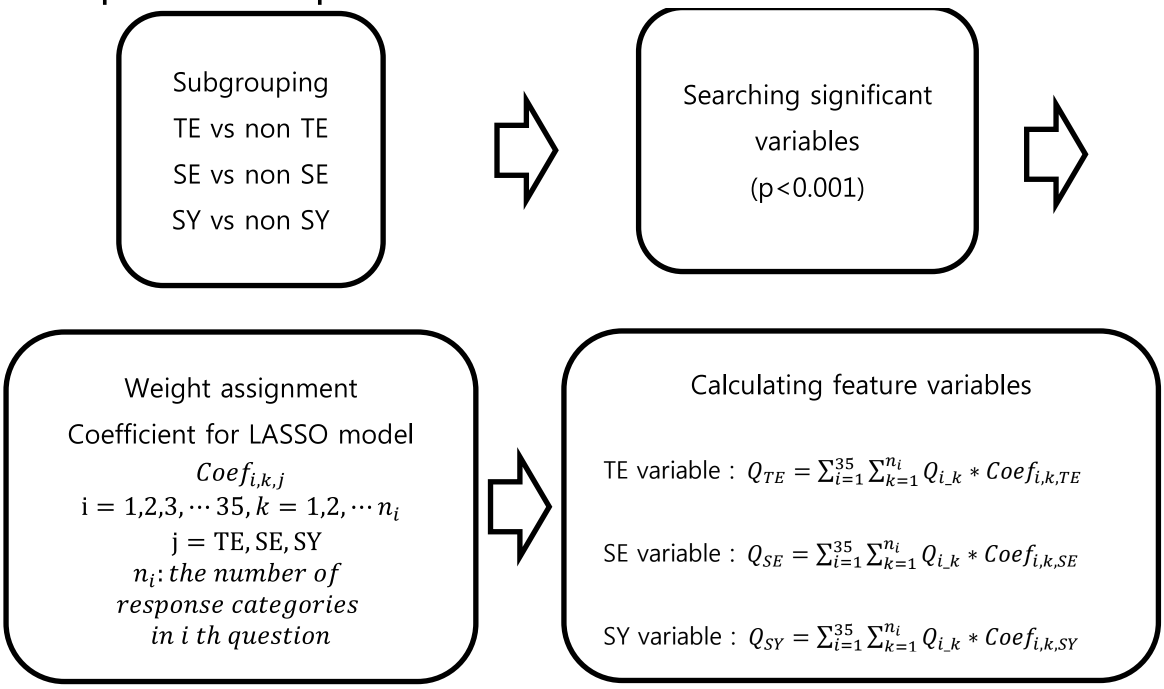

Supplement: Supplementary file 1 — Table S1. List of Questions. Table S2. Result of estimated coefficients for Facial features for each SC type using LASSO. Table S3. Result of estimated coefficients for Voice features for each SC type using LASSO. Table S4. Result of estimated coefficients for Body shape features for each SC type using LASSO. Table S5. Result of estimated coefficients for Questionnaire features for each SC type using LASSO. Table S6. Result of estimated coefficients for Questionnaire continuous features for each SC type using OLS. Table S7. Result of estimated coefficients for estimated probability of individual diagnostic model for each SC type using LASSO. Figure S1. The procedure of generating continuous variables with the response categories of the questions in the questionnaire. [file 9180159.f1.pdf]
